# sEMG-Based Trunk Compensation Detection in Rehabilitation Training

**DOI:** 10.3389/fnins.2019.01250

**Published:** 2019-11-21

**Authors:** Ke Ma, Yan Chen, Xiaoya Zhang, Haiqing Zheng, Song Yu, Siqi Cai, Longhan Xie

**Affiliations:** ^1^School of Mechanical and Automotive Engineering, South China University of Technology, Guangzhou, China; ^2^Shien-Ming Wu School of Intelligent Engineering, South China University of Technology, Guangzhou, China; ^3^Department of Rehabilitation Medicine, The Third Affiliated Hospital, Sun Yat-sen University, Guangzhou, China

**Keywords:** trunk compensation detection, surface electromyography, stroke, rehabilitation training, support vector machine

## Abstract

Stroke patients often use trunk to compensate for impaired upper limb motor function during upper limb rehabilitation training, which results in a reduced rehabilitation training effect. Detecting trunk compensations can improve the effect of rehabilitation training. This study investigates the feasibility of a surface electromyography-based trunk compensation detection (sEMG-bTCD) method. Five healthy subjects and nine stroke subjects with cognitive and comprehension skills were recruited to participate in the experiments. The sEMG signals from nine superficial trunk muscles were collected during three rehabilitation training tasks (reach-forward-back, reach-side-to-side, and reach-up-to-down motions) without compensation and with three common trunk compensations [lean-forward (LF), trunk rotation (TR), and shoulder elevation (SE)]. Preprocessing like filtering, active segment detection was performed and five time domain features (root mean square, variance, mean absolute value (MAV), waveform length, and the fourth order autoregressive model coefficient) were extracted from the collected sEMG signals. Excellent TCD performance was achieved in healthy participants by using support vector machine (SVM) classifier (LF: accuracy = 94.0%, AUC = 0.97, F1 = 0.94; TR: accuracy = 95.8%, AUC = 0.99, F1 = 0.96; SE: accuracy = 100.0%, AUC = 1.00, F1 = 1.00). By using SVM classifier, TCD performance in stroke participants was also obtained (LF: accuracy = 74.8%, AUC = 0.90, F1 = 0.73; TR: accuracy = 67.1%, AUC = 0.85, F1 = 0.71; SE: accuracy = 91.3%, AUC = 0.98, F1 = 0.90). Compared with the methods based on cameras or inertial sensors, better detection performance was obtained in both healthy and stroke participants. The results demonstrated the feasibility of the sEMG-bTCD method, and it helps to prompt the stroke patients to correct their incorrect posture, thereby improving the effectiveness of rehabilitation training.

## Introduction

Stroke is one of the leading causes of disability in the world (Burton et al., 2018), and approximately 80% of stroke patients have accompanying upper limb motor dysfunction (such as muscle weakness, abnormal posture control, and abnormal limb coordinated exercise). Upper limb motor dysfunction seriously affects a stroke patient’s daily life and work (Hatem et al., 2016). Many clinical practices show that rehabilitation training can effectively promote the recovery of upper limb motor dysfunction (Zhang et al., 2015). However, during rehabilitation training, patients often compensate for the impaired upper limb by recruiting intact trunk muscles and joints (Cirstea and Levin, 2000). This compensatory motion is called trunk compensation. According to the different motion characteristics of the trunk, there are three common trunk compensations: lean-forward (LF), trunk rotation (TR), and shoulder elevation (SE) (Dolatabadi et al., 2017). Regardless of the type of compensation, the trunk compensation reduces the effect of rehabilitation training and hinders the recovery of upper limb motor dysfunction (Levin et al., 2009).

To improve the effectiveness of rehabilitation training, measures should be taken to detect trunk compensations. In early studies, physical constraints (Michaelsen and Levin, 2004; Pain et al., 2015; Greisberger et al., 2016) were applied to the stroke patient’s trunk using straps or special wire harnesses, restraining the patient’s trunk on the chair without compensation. These limitations on trunk compensation can improve the arm function of the patient (Wee et al., 2014). However, rehabilitation training for patients is repetitive and intensive. Long-term physical constraints can cause discomfort and anxiety. In addition, once the rehabilitation training exceeds the patient’s range of motion, it is highly likely that strain to the patient will result. Therefore, without the restraint of the trunk, detecting trunk compensations by detection technology is more suitable and effective for stroke patients.

At present, wearable inertial sensors (Najafi et al., 2003) or cameras (Bakhti et al., 2018) are mainly used to detect trunk compensations. Although wearable inertial sensor systems are often used to assess and monitor upper limb motor ability in stroke patients (Zhang et al., 2012; Urbin et al., 2015), a preliminary study shows that compensation strategies can be identified by inertial sensors (Salazar et al., 2014). For instance, Ranganathan et al. (2017) used two wearable inertial sensors to collect motion data for 20 healthy participants when simulating trunk compensations. Using the naïve Bayesian classifier for binary classification (whether there is trunk displacement), the authors obtained an accuracy of 88.6%. In short, the trunk compensation detection (TCD) method based on inertial sensors achieves unsatisfactory detection accuracy (<90%) and lacks TCD in stroke patients. In addition, in order to reduce the measurement errors, some actions, such as arm horizontal abduction, are needed to calibrate the inertial sensors, but it is difficult for stroke patients to perform these actions. Besides, the position of the inertial sensors may change during motion due to the flexibility of human skin causing the reduction of the effectiveness of data acquisition.

Currently, camera-based detection method has gained wide popularity (Duff et al., 2010; Subramanian et al., 2013). For example, Taati et al. (2012) used a depth camera to capture video data from seven healthy participants simulating LF, TR, SE, and slouch compensation. Using an improved hidden Markov support vector machine (HM-SVM) classifier for multiclassification, the authors achieved an average accuracy of 85.9% per frame. Subsequently, Zhi et al. (2017) captured video data of not only simulated LF, TR, and SE compensation for 10 healthy participants but also actual trunk compensations for 9 stroke participants with a Kinect v2 camera. Using an SVM and recurrent neural network (RNN) classifier, the authors achieved similar classification performance. In the simulated trunk compensation dataset of healthy participants, the detection performance of LF compensation was the highest (AUC = 0.98, F1 = 0.82), followed by TR compensation (AUC = 0.77, F1 = 0.57), and finally SE compensation (AUC = 0.66, F1 = 0.07). In contrast, lower detection performance was achieved in the actual trunk compensation dataset of stroke patients, namely: LF compensation (AUC = 0.77, F1 = 0.17), TR compensation (AUC = 0.81, F1 = 0.27), and SE compensation (AUC = 0.27, F1 = 0.07). We found that the detection performance is not ideal, especially in the detection of stroke participants’ trunk compensations. In addition, a camera-based detection system is limited to indoor environments due to dependence on illumination. What’s more, a camera-based detection system can lead to privacy issues, especially in regard to stroke patients. Because of the shortcomings of the above two methods, a convenient, environment-independent, and accurate detection method is needed to detect trunk compensations.

Surface electromyography (sEMG) signal is a bioelectrical signal containing muscle motion information. Compared to cameras and inertial sensors, the acquisition of sEMG signals does not depend on an external environment, such as illumination, nor does it require calibration. Based on these advantages, sEMG-based pattern recognition technology emerged and developed rapidly. Feature extraction and classification are the most critical technologies in sEMG-based pattern recognition technology. To date, the time domain, frequency domain, and time-frequency domain features have been widely used for the analysis and processing of sEMG signals (Burhan and Ghazali, 2016; Majid et al., 2018; Phinyomark et al., 2018). In addition, many classifier algorithms have appeared for classification, such as SVM, artificial neural networks (ANNs), and linear discriminant analysis (LDA) (Chowdhury et al., 2013; Nazmi et al., 2016). Due to a variety of features and classifiers, the sEMG-based pattern recognition technology has been widely used for upper limb motion pattern recognition (Lucas et al., 2008; Yang and Chen, 2016; Lu et al., 2017) and upper limb continuous motion estimation (Liu et al., 2017; Zhang et al., 2017). However, it has not yet been used in TCD.

Therefore, in this paper, the sEMG-based TCD (sEMG-bTCD) method is proposed and its feasibility is verified by experiments. The experiment was divided into two sessions. First, five healthy participants were recruited to verify the practical feasibility of the method, and then nine stroke participants were recruited to verify the clinical feasibility. Specifically, we selected nine trunk muscles from the trunk muscles that control the three trunk compensations and collected sEMG signals from these muscles. Then, we extracted five time domain features from the acquired sEMG signals, and performed the TCD by using SVM classifier, and achieved excellent detection performance. The rest of this article is structured as follows. The section “Materials and Methods” introduces the participants and experimental protocols. The section “Trunk Compensation Detection Procedure” provides the TCD procedure in detail, specifically, an improved active segment detection method. The section “Results and Discussion” analyzes the experimental results and discussion. Finally, the section “Conclusion” summarizes the paper.

## Materials and Methods

### Participants

For this paper, 14 participants were recruited to participate in the experiment, including five healthy subjects (all male, age 25.7 ± 1.8 years, without upper limb motor dysfunction) and nine stroke subjects. Ethics approval and consent to participate (i.e., informed consent) was obtained from all the participants to complete the protocol approved by the Guangzhou First People’s Hospital Department of Ethics Committee. All the research was performed in accordance with the Declaration of Helsinki. Stroke subjects were screened by a rehabilitation therapist. The inclusion criteria for stroke subjects included: (a) between the ages of 20 and 80 years; (b) the Brunnstrom Scale above stage II to have upper limb exercise capacity; and (c) having cognitive and comprehension skills. Finally, nine stroke patients were recruited to participate in the experiment, as detailed in Table 1.

**TABLE 1:** Details of the nine stroke subjects.

**Subject**	**Age (years)**	**Time since stroke (months)**	**BS**	**FMA-UE**
S1	45–50	17	II	30
S2	50–55	7	II	9
S3	50–55	6	II	15
S4	50–55	11	II	15
S5	70–75	5	III	27
S6	50–55	4	II	26
S7	25–30	14	II	22
S8	65–70	1	IV	38
S9	45–50	25	III	48

*BS, Brunnstrom Scale, the stage level from I (no movement) to VI (approximately normal coordinated movement); FMA-UE, Fugl-Meyer Assessment Upper Extremity, scores from 0 (no movement) to 66 (normal movement).*

### Experimental Protocols

#### Rehabilitation Training Tasks and Trunk Compensations

**FIGURE 1:**
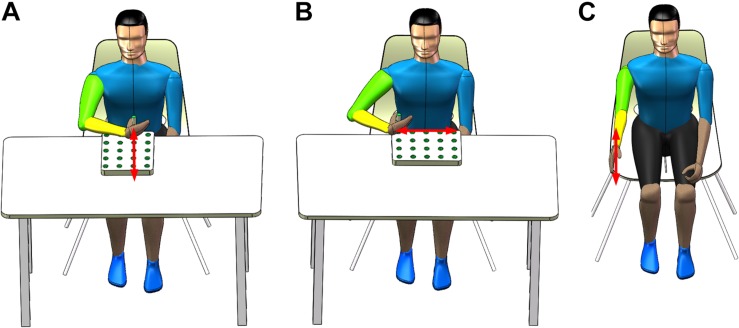
Three rehabilitation training tasks. **(A)** Reach-forward-back, **(B)** reach-side-to-side, and **(C)** reach-up-to-down.

Each participant performed three basic rehabilitation training tasks, including the reach-forward-back (T1), reach-side-to-side (T2), and reach-up-to-down (T3) motions. The T1 motion refers to the straight forward and backward movement of an upper limb (e.g., right hand, left hand symmetrical to right hand) in the sagittal and transverse plane. The starting point is located on the central axis of the human body, 20 cm away from the participant as shown in Figure 1A. The range of motion is the distance (approximately 24 cm) between the center of the five circular grooves of the wooden flashboard (34 cm × 28 cm × 2 cm). The T2 motion means that an upper limb moves in a straight line with adduction and abduction in the transverse plane. The starting point is located on the side of the participant’s body, 20 cm away from the participant as shown in Figure 1B. The range of motion is also 24 cm. The T3 motion is a shoulder flexion with a range of 0 to the maximum angle of the participant (<180°) in the sagittal plane, as shown in Figure 1C.

**FIGURE 2:**
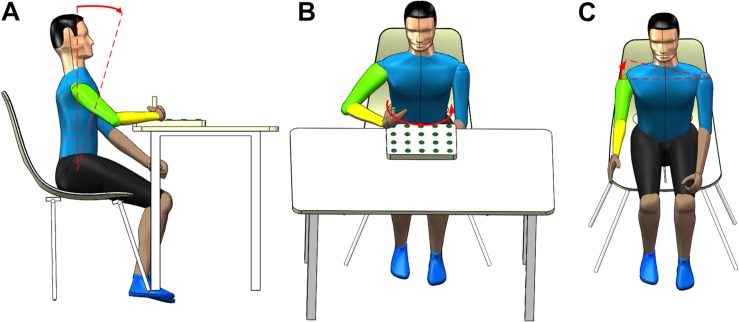
Three common types of trunk compensation. **(A)** shoulder elevation, **(B)** lean-forward, and **(C)** trunk rotation.

The rehabilitation training tasks involve the shoulder and elbow joints, which contribute to the recovery of the motor function of these two joints. More importantly, these tasks aim to elicit three common trunk compensations: LF, TR, and SE. LF compensation happens when a participant’s hip bending angle is <90°, as shown in Figure 2A. TR compensation happens when a participant rotates his trunk in the transverse plane, as shown in Figure 2B. SE compensation happens when a participant raises his unilateral shoulder in the coronal plane, as shown in Figure 2C. Basic motions (tasks) correspond to trunk compensations. The participant may experience LF compensation when performing the T1 motion. The participant may experience TR compensation when performing the T2 motion. In addition, the participant may experience SE compensation when performing the T3 motion.

#### sEMG Acquisition System

**FIGURE 3:**
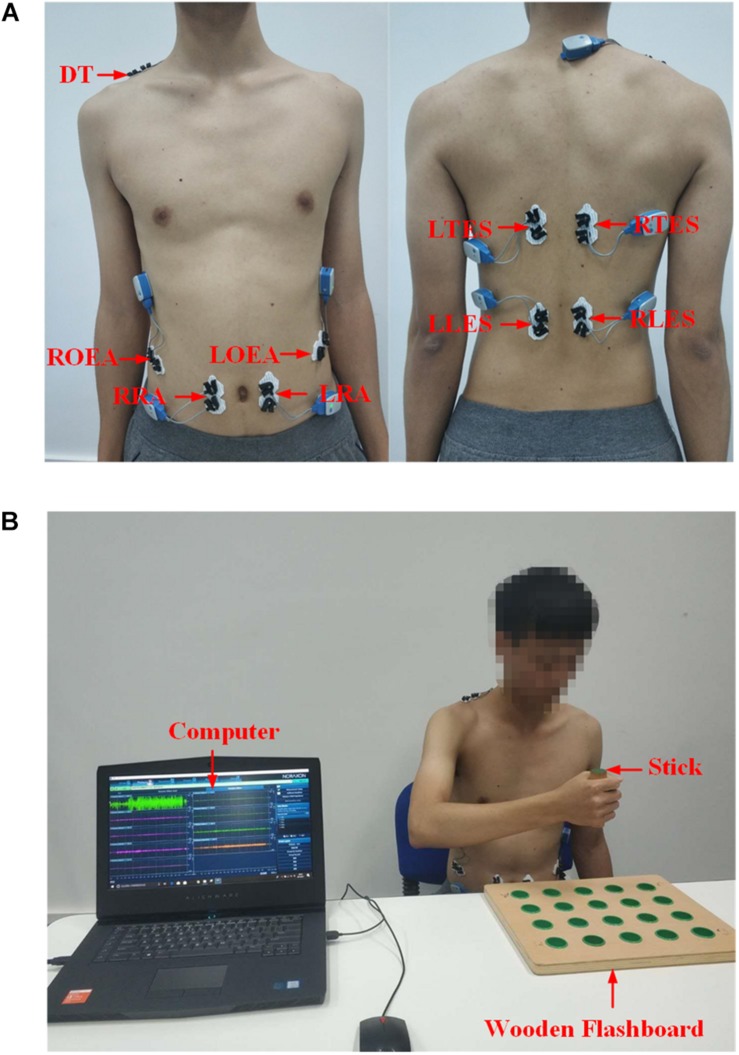
**(A)** Electrodes placement on trunk muscles. DT, descending part of trapezius; LRA, left rectus abdominis; RRA, right rectus abdominis; LOEA, left obliquus externus abdominis; ROEA, right obliquus externus abdominis; LTES, left thoracic erector spinae; RTES, right thoracic erector spinae; LLES, left lumbar erector spinae; RLES, right lumbar erector spinae. **(B)** A snapshot of the experiment setup.

The collection of the sEMG signals is strictly in accordance with the recommended standards (Hermens et al., 1999; Konrad, 2005). Combining some references (Larivière et al., 2000; Ghofrani et al., 2017; Mueller et al., 2017; Varrecchia et al., 2018) and the physiology, nine superficial trunk muscles were selected from the numerous trunk muscles involved in the three trunk compensations. These muscles are the left and right rectus abdominis (LRA and RRA), the left and right obliquus externus abdominis (LOEA and ROEA), the left and right thoracic erector spinae (LTES and RTES), the left and right lumbar erector spinae (LLES and RLES), and a descending part of the trapezius (DT, on the side of the upper limb of the motion). The DT muscle plays a major role in the SE compensation. The LOEA and ROEA muscles play a key role in the TR compensation, while other muscles help to control the LF compensation. Then, nine pairs of surface electrodes were used to record the sEMG signals of the nine trunk muscles. The surface electrode material was AgCl, and the distance between the electrodes was 2 cm. The direction of the electrodes was parallel to the muscle fibers. The electrodes were placed as shown in Figure 3A. The surface electrodes for the LRA and RRA were placed 2 cm left and right next to the umbilicus. The surface electrodes for the LOEA and ROEA were placed 15 cm left and right next to the umbilicus. The surface electrodes for the LTES and RTES were placed 3 cm left and right of the T10 spinous process. The surface electrodes for the LLES and RLES were placed 3 cm left and right of the L3 spinous process (Larivière et al., 2000; Ghofrani et al., 2017). Prior to placing the surface electrodes, we wiped alcohol on the skin surface to reduce skin impedance. Then, the 1st–9th channels of the 16-channel Ultium-EMG sensor system (Noraxon USA Inc., Scottsdale, AZ, United States) with a sampling frequency of 2000 Hz were used to collect the original sEMG signals. With the amplitude range of 100–5000 μV and the frequency component of 0–500 Hz (Merletti et al., 1992), the sEMG signals were amplified 1000 times and filtered a 10–500 Hz bandpass.

#### Experimental Sessions

The experimental protocols consisted of two sessions, which included the healthy group and the stroke group experiment sessions. First, we investigated the feasibility of the sEMG-bTCD method with the simulated trunk compensations of the healthy group. Second, we verified whether the proposed method could detect the actual trunk compensations in stroke patients. There are two reasons for using healthy group simulation data rather than data obtained directly from stroke patients. On the one hand, this is a novel study that cannot be used directly on stroke patients. On the other hand, a previous study (Zhi et al., 2017) has shown that healthy people can obtain valuable experimental data by simulating trunk compensations.

The experimental setup is shown in Figure 3B. Each participant in the healthy group sat on the chair without any physical restraint on their trunk. A horizontal table was placed in front of the participants. A wooden flashboard was fixed on the table to guide the participant’s motions. With a stick, the participant completed three tasks (T1, T2, and T3 motions) on the wooden flashboard at a normal speed. Additionally, the participants simulated three types of trunk compensations (LF, TR, and SE compensation) according to the guidance and demonstration of our research team. Unlike the healthy group, all participants in the stroke group performed three tasks with both the healthy and affected hands. The data from the healthy hand performing tasks were used as the data with no compensation. The data from the affected hand performing tasks represented the trunk compensation data. Each motion was repeated 10 times. To prevent fatigue, each participant rested for 10 s between the two motions and rested for 1 min after the five motions. At least one rehabilitation therapist participated in the entire experiment of the stroke group, helping our research team visually observe whether stroke patients developed trunk compensation and the type of trunk compensation.

## Trunk Compensation Detection Procedure

**FIGURE 4:**
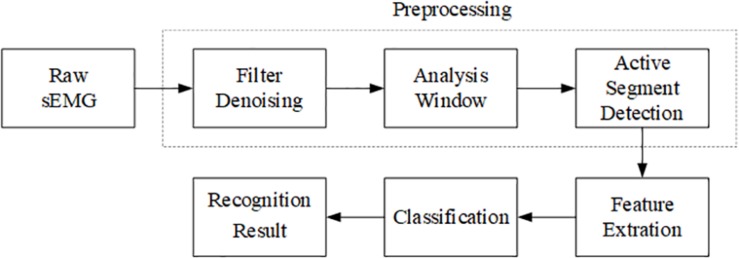
Flowchart of the procedures for sEMG processing.

The processing of the sEMG signals was implemented with MATLAB 2017a (The MathWorks Inc., Natick, MA, United States) (Figure 4), including preprocessing, feature extraction, and classification. The preprocessing consists of three parts: filter denoising, analysis window, and active segment detection.

### Preprocessing

#### Filter Denoising

Various external factors during the acquisition process, such as 50 or 60 Hz power frequency interference, motion artifact, and ECG interference can easily interfere with the sEMG signals (Phinyomark et al., 2012; Barrios-Muriel et al., 2016). To eliminate ECG interference and motion artifacts, a 20–200 Hz bandpass filtering for sEMG signals was implemented by using a Butterworth filter with 0.1 dB passband ripple and 50 dB stopband attenuation. A 50 Hz notch filter was implemented using a Butterworth filter to eliminate power frequency interference. These preprocessing methods were intended to improve the signal-to-noise ratio (SNR) of the sEMG signals.

#### Analysis Window

Due to the randomness and non-stationarity of the sEMG signals, the analysis window, rather than the instantaneous value, is a useful input in the pattern recognition process (Smith et al., 2010). In this paper, we used an overlap analysis window with a window length of 256 ms (512 samples) and a window sliding step size of 64 ms (128 samples). So, the sEMG signals collected in 1 s can be divided into 12 analysis windows. The subsequent active segment detection, feature extraction, and classification in this paper were based on these analysis windows.

#### Active Segment Detection

In this paper, a sample entropy (SampEn) method based on optimal threshold was proposed to detect the active segments of sEMG signals. The SampEn is an improved method for measuring the complexity of time series based on approximate entropy (ApEn) (Richman and Moorman, 2000). A study has applied SampEn based on a fixed threshold to the active segmentation of sEMG signals (Zhou and Zhang, 2013). The fixed threshold is an empirical value determined by experiments. However, it is very difficult to choose a general fixed threshold for different participants or motions. Therefore, we proposed a SampEn method based on an optimal threshold. The implementation was divided into three steps: calculating SampEn, detecting active segments based on a fixed threshold, and calculating optimal threshold.

In the first step, the SampEn of an analysis window (*M* samples, *M=512*) is calculated. The time sequence sEMG_sum_(*k*) of the sum of 9-channel signals is constructed as follows:

(1) $${\text{sEMG}}_{\text{sum}}\,\left(k\right)=\sum_{i=1}^{C}{\text{sEMG}}_{i}\,\left(k\right)$$

where *C* is the total number of channels (*C=9*), *i* is the channel number, and *k* is the number of points in the analysis window.

Then, the scalar time series sEMG_sum_(*k*), *k* = 1, 2, …, *M*, are embedded in the delayed *m*-dimensional space to form a set of *m*-dimensional vectors (a data segment of length *m*) (Zhang and Zhou, 2012; Yentes et al., 2013):

(2) $$\left\{ \begin{array}{c} {\text{sEMG}}_{\text{sum}}^{m}(j)={\left[{\text{sEMG}}_{\text{sum}}^{m}(j+p)\right]}_{p=0}^{m-1}\\ j=1,2,\ldots ,\text{M-m}+1 \end{array}\right.$$

The probability B^*m*^(*r*) of two sequences matching *m* points is computed by calculating the average number of vector pairs whose distance is lower than the similar tolerance *r*. Similarly, the probability A^*m*^(*r*) of the *m+1* dimension can be calculated. Finally, the SampEn is calculated as:

(3) $$\text{SampEn}\,\left(m,r,M\right)=-\text{ln}\,\left(\frac{{\text{A}}^{m}\,\left(r\right)}{{\text{B}}^{m}\,\left(r\right)}\right)$$

The choice of the dimension *m* and the similar tolerance *r* determines the calculation result of SampEn. There are empirical formulas for these values, which are (Pincus, 1991; Costa et al., 2002): *m* = 1 or 2, *r* = (0.15–0.25) ^∗^ σ. Where σ is the standard deviation of the entire data sequence sEMG_sum_. In this paper, these values are: *m* = 2, *r* = 0.25^∗^ σ.

In the second step, active segment detection based on a fixed threshold is performed. According to the first step, the SampEn(*l*) of the *l*th analysis window is obtained. Then, the state function *s*(*l*) of the *l*th analysis window is calculated:

(4) $$s\left(l\right)=\{\begin{array}{c} 0,\text{SampEn}\left(l\right)<\text{Th}\\ 1,\text{SampEn}\left(l\right)\geq \text{Th} \end{array}$$

where Th is the fixed threshold value. The condition of an active segment based on the state function is:

(5) $$\{\begin{array}{c} s(l_{1}-1)=0\text{and}s(l_{1})=1\\ s\left(l_{2}-1\right)=1\text{and}s(l_{2})=0\\ L=l_{2}-l_{1}\geq L_{0}=12\times \text{sec} \end{array}$$

where *l*_*1*_ and *l*_*2*_ are the starting and ending analysis windows of an active segment, *L* is the number of analysis windows between *l*_*1*_ and *l*_*2*_, sec means time sec seconds, and *L*_*0*_ is the preset number of analysis windows in an active segment. Only when *L* is not less than *L*_*0*_ can this active segment be considered as an effective active segment. Otherwise, this active segment is still regarded as a noise. In addition, multiple active segments (such as *n*) may be detected in the acquired sEMG signals. In order to distinguish each active segment, the *l*_*1*_ and *l*_*2*_ of each active segment are stored in one-dimensional arrays *x*_*1*_ and *x*_*2*_ of length *n*, respectively.

**FIGURE 5:**
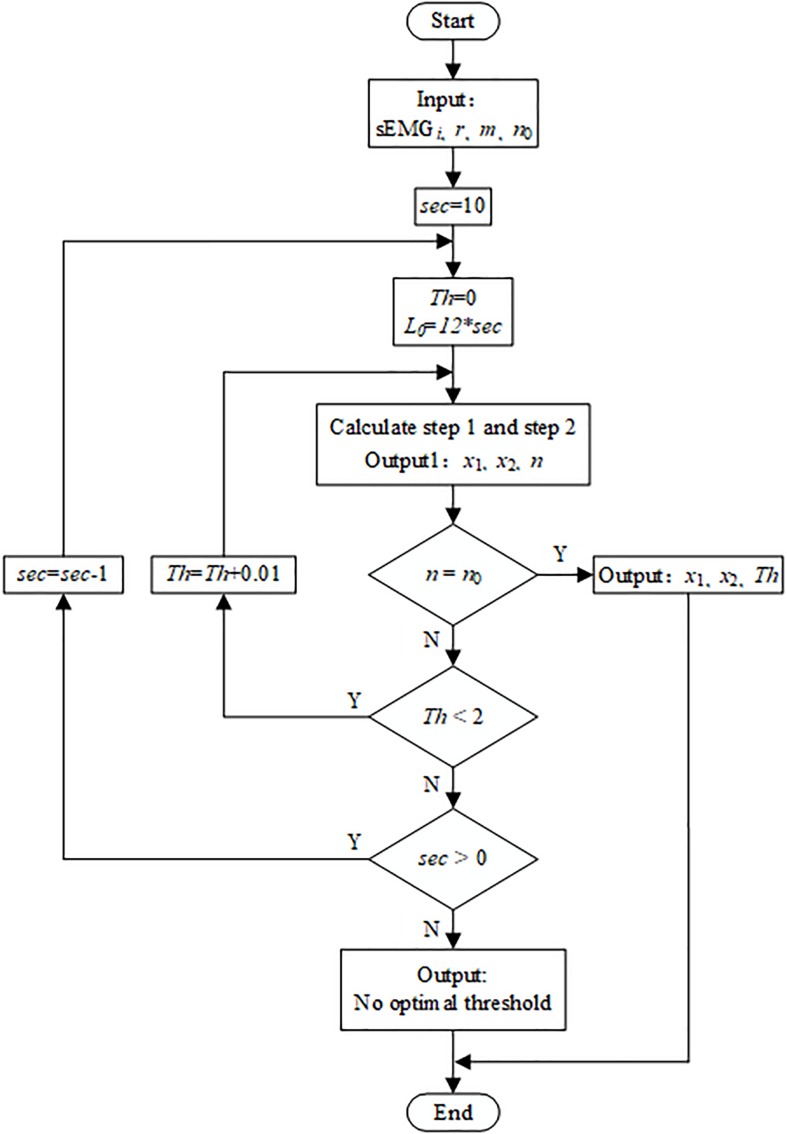
Flowchart for calculating the optimal threshold.

In the third step, the optimal threshold is calculated by iteration. The objective function is not only to accurately detect the known *n*_*0*_ active segments (*n*_0_ = 5 in this paper), but also to make each active segment as long as possible so as to contain more motion information. Therefore, the objective function is realized by two loops, and the flowchart is shown in Figure 5.

**FIGURE 6:**
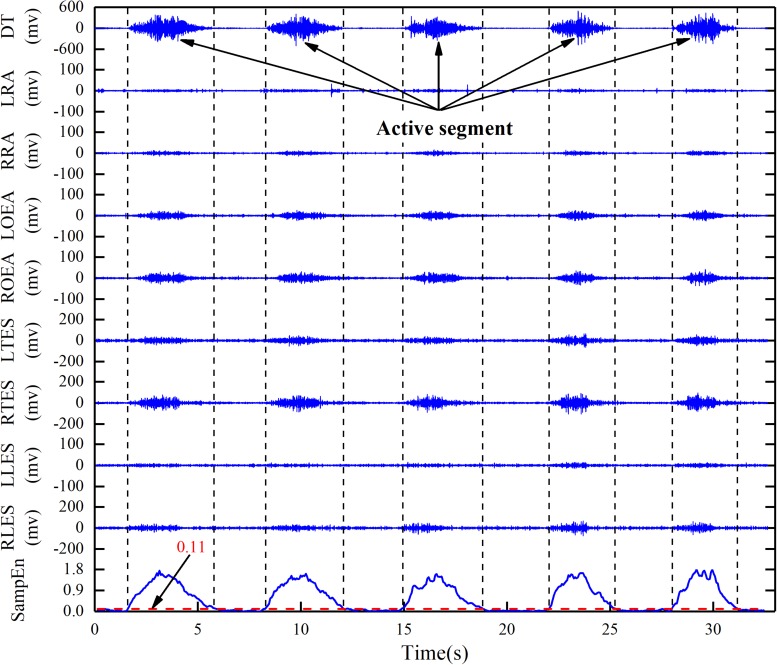
Active segment detection based on adaptive SampEn threshold algorithm in reach-up-to-down motion. DT, descending part of trapezius; LRA, left rectus abdominis; RRA, right rectus abdominis; LOEA, left obliquus externus abdominis; ROEA, right obliquus externus abdominis; LTES, left thoracic erector spinae; RTES, right thoracic erector spinae; LLES, left lumbar erector spinae; RLES, right lumbar erector spinae.

The loop variables of the outer loop and the inner loop are, respectively, the number *L*_*0*_ of the analysis windows in the active segment (replacing *L*_*0*_ with sec, 10≥sec≥1) and the SampEn threshold Th (0≤Th≤2). The loop body performs the first two steps in sequence, and outputs the number *n* of detected active segments and determines whether it is equal to *n*_*0*_. If *n* is equal to *n*_*0*_, the *x*_*1*_, *x*_2_, and Th values at the moment are output. Taking a stroke participant performing reach-up-to-down motion as an example, the active segment detection result of 9-channel sEMG signals was shown in Figure 6.

### Feature Extraction

Compared with the frequency domain and time-frequency domain features, the time domain features are simple and less time-consuming. So, we selected five commonly used time domain features to establish feature vectors. These features are root mean square (RMS), variance (Varrecchia et al., 2018), MAV, waveform length (WL), and the fourth order autoregressive model coefficient (4th-ARMC).

The RMS is the square root of the average power of the sEMG signals in a given analysis window. It is calculated as:

(6) $${\text{RMS}}_{i}\,\left(t\right)=\sqrt{\frac{1}{M}\,\sum_{k=1}^{M}{\text{sEMG}}_{i}^{t}\,{\left(k\right)}^{2}}$$

where *i* is the channel number (*i* = 1, 2, 3, …, 9), *t* is the analysis window number, *k* is the number of points in the *t*th analysis window.

The VAR reflects the extent to which the sEMG signal deviates from the average and is calculated as:

(7) $$\left\{ \begin{array}{c} {\text{AVR}}_{i}\,\left(t\right)=\frac{1}{M}\,\sum_{k=1}^{M}{\text{sEMG}}_{i}^{t}\,\left(k\right)\\ {\text{VAR}}_{i}\,\left(t\right)=\frac{1}{M}\,\sum_{k=1}^{M}{\left({\text{sEMG}}_{i}^{t}\,\left(k\right)-{\text{AVR}}_{i}\,\left(t\right)\right)}^{2} \end{array}\right.$$

In statistics, the sEMG signal is approximated as a random signal with a mean of zero. The average value does not reflect the signal characteristics. Therefore, the absolute value of the sEMG signal is averaged, which is the definition of MAV. It is calculated as:

(8) $${\text{MAV}}_{i}\,\left(t\right)=\frac{1}{M}\,\sum_{k=1}^{M}\left|{\text{sEMG}}_{i}^{t}\,\left(k\right)\right|$$

The WL is a simple accumulation of sEMG signal lengths that can reflect the complexity of the sEMG signal waveform. It is defined as:

(9) $${\text{WL}}_{i}\,\left(t\right)=\sum_{k=1}^{M}\left|{\text{sEMG}}_{i}^{t}\,\left(k+1\right)-{\text{sEMG}}_{i}^{t}\,\left(k\right)\right|$$

The AR model is a linear model used for time-series analysis of sEMG signals. It is defined as:

(10) $${\text{sEMG}}_{i}^{t}\,\left(k\right)=\sum_{j=1}^{q}a_{j}\,{\text{sEMG}}_{i}^{t}\,\left(k-j\right)+e_{i}^{t}\,\left(k\right)$$

where *q* is the order of AR model (*q=4*), *a*_*j*_ is the *j*th order AR coefficient, and $e_{i}^{t}\,\left(k\right)$ is the white noise residual.

The five time domain features were extracted from each analysis window in a single channel sEMG signal. In this paper, 9-channel sEMG signals were collected, so 1 ^∗^ 45 (5 ^∗^ 9) one-dimensional feature vector was extracted from each analysis window. However, the stroke patient’s affected side may be left hand or right hand. Different affected sides lead to SE and TR compensations in opposite directions. This results in the opposite effect of the four pairs of muscles (LRA/RRA, LOEA/ROEA, LTES/RTES, and LLES/RLES). Correspondingly, the consistency of feature vectors extracted from the posterior 8-channel sEMG signals is poor or even contrary, which is not conducive to classification. Therefore, in order to reduce the influence of different affected hands, we reconstructed five time domain features of the posterior 8-channel sEMG signals as:

(11) $$\left\{ \begin{array}{c} \begin{array}{c} {\text{feat}}_{i}^{\prime }\,\left(t\right)={\text{feat}}_{i}\,\left(t\right)+{\text{feat}}_{i+1}\,\left(t\right)\\ {\text{feat}}_{i+1}^{\prime }\,\left(t\right)=\text{abs}\,\left({\text{feat}}_{i}\,\left(t\right)-{\text{feat}}_{i+1}\,\left(t\right)\right) \end{array}\\ i=2,4,6,8 \end{array}\right.$$

where *i* is the *i*th channel, *t* is the analysis window number, and feat_*i*_(*t*) stands for any of the above five time domain features (RMS, VAR, MAV, WL, and 4th-ARMC). Feature reconstruction only changed the feature values, but did not change the feature dimension, so each analysis window extracted 1 ^∗^ 45 dimension feature vector. In addition, considering the convenience of the method in clinical application, the number of four pairs of trunk muscles was continuously reduced, and finally 9, 7, 5, and 3 channels were investigated. Correspondingly, the feature vector dimensions extracted by each analysis window are 1 ^∗^ 45, 1 ^∗^ 35, 1 ^∗^ 25, and 1 ^∗^ 15, respectively.

### Classification

As the SVM algorithm implements the principle of structural risk minimization (Burges, 1998), it has unique advantages in solving small sample, non-linear, and high-dimensional pattern recognition. Some studies (e.g., Bellingegni et al., 2017; Quitadamo et al., 2017) have also shown that the SVM has higher classification performance. Therefore, we choose the SVM classifier. The purpose of the SVM is to find an optimal hyperplane to segment samples. The principle of segmentation is to maximize the interval and finally transform it into a convex quadratic programming problem (Scholkopf and Smola, 2001), expressed as:

(12) $$\left\{ \begin{array}{c} \text{min}\,\frac{1}{2}\,{||w||}^{2}\\ s.t.y_{t}\,\left(w^{*}\,x_{t}+b\right)-1\geq 0 \end{array}\right.$$

where (*x*_*t*_,*y*_*t*_) is the *t*th data point and (*w*, *b*) is the hyperplane parameter. The Lagrange multiplier is used to solve the problem.

The TCD model is a four-class model (NC, LF, TR, and SE), so the one-versus-one strategy is used for multiclassification. And we used the LIBLINEAR (Fan et al., 2008) toolkit for SVM classifier. When using L2-regularized L2-loss support vectorclassification, only the penalty factor *C* needs to be searched (Hsu et al., 2003). In this paper, cross-validation was used to adjust parameter *C* from small to large. When the increase *C* did not change the classification result much, the debugging was finished and the relatively small *C* value was selected to improve the convergence speed of the model (finally, *C* = 1). In addition, the hold-out method was used to evaluate classifier performance. Specifically, in order to maintain the consistency of data distribution as much as possible, a training subset and a test subset were randomly divided into 80:20% of each class’s feature set. The training subsets and test subsets of the four classes were, respectively, combined to form a training set and a test set. In addition, we used the 100-time hold-out method to obtain a stable and reliable evaluation result. Also, we chose classification accuracy, F1-score, receiver operating characteristic (ROC) curve and the under the curve (AUC) as model evaluation parameters. Classification accuracy is the most commonly used classification model evaluation index, which refers to the ratio of the number of correctly classified samples to the total sample. The F1-score is the harmonic mean of precision and recall. The ROC curve is an evaluation curve in which the false positive rate (FPR) is the horizontal axis and the true positive rate (TPR) is the vertical axis. The AUC is defined as the area enclosed by the ROC curve and the coordinate axis.

## Results and Discussion

### Results

#### Classification Accuracy

**FIGURE 7:**
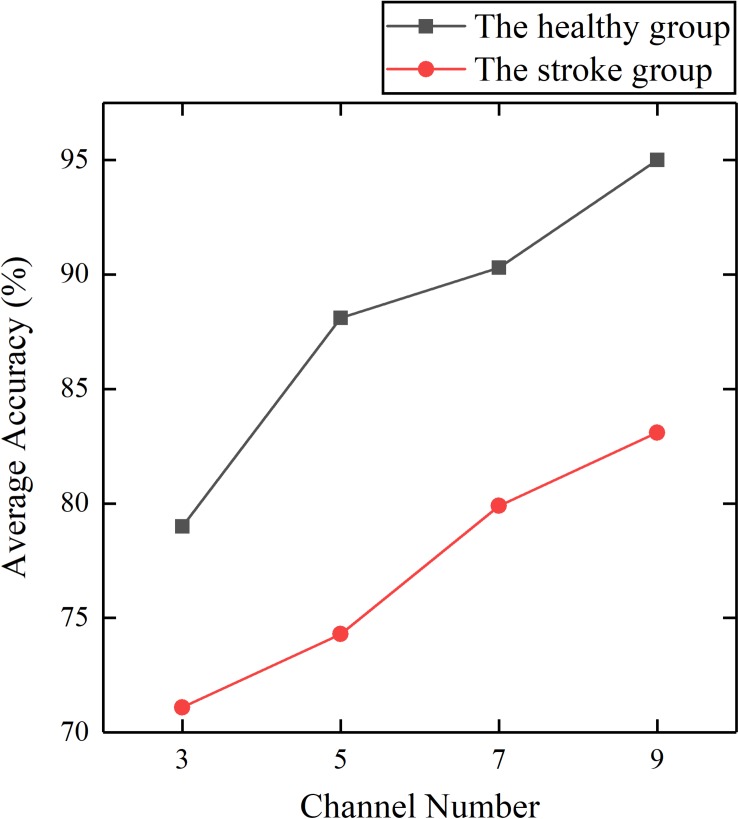
Average accuracy of the healthy and stroke group models established by different channel numbers.

To investigate the feasibility of the proposed method, we established two trunk compensation models based on the healthy group dataset and the stroke group dataset. First, the impact of the number of channels on the classification was investigated. It can be seen from Figure 7 that fewer channels result in lower average accuracy. Based on the principle of optimal accuracy, only the detailed detection results of nine channels were reported below.

**FIGURE 8:**
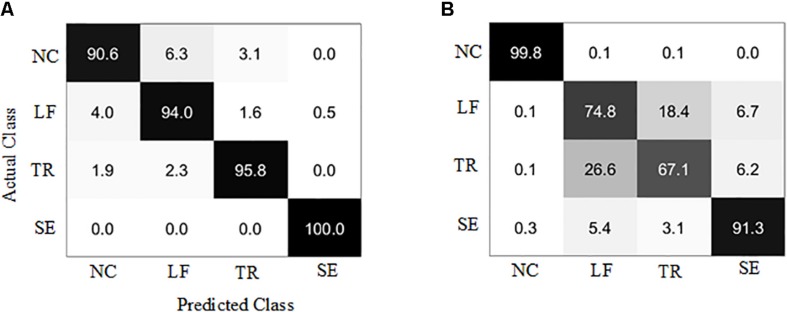
Confusion matrix of the trunk compensation detection models. **(A)** Healthy group. **(B)** Stroke group. NC, no compensation; LF, lean-forward; TR, trunk rotation; SE, shoulder elevation.

The results showed that the healthy group model and the patient group model achieved an average accuracy of 95.0 and 83.1% (Figure 7). The confusion matrices of the two models were given as grayscale images (Figure 8). The diagonal elements (*n*, *n*) (*n* = 1, 2, 3, 4) in the confusion matrix represent the classification accuracy of each class, while the other elements represent the error classification rate.

#### ROC and F1-Score

**FIGURE 9:**
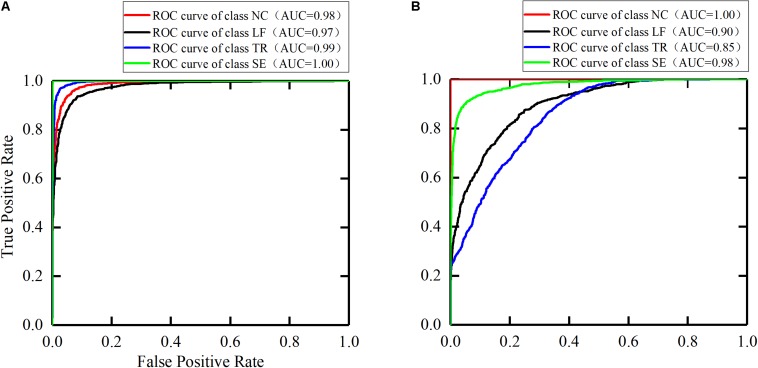
The ROC curves for the trunk compensation detection models. **(A)** The healthy group. **(B)** The stroke group. NC, no compensation; LF, lean-forward; TR, trunk rotation; SE, shoulder elevation.

Due to the difference of each participant’s motor function, the sample distribution of the four classes is slightly unbalanced. Therefore, we also selected F1-score, ROC, and AUC as model evaluation parameters. These parameters are often used for binary classification of unbalanced distribution. However, the models established in this paper were four-class models, so the conversion was needed to obtain these parameters. We considered the current class as the positive class, and the remaining classes as the negative class. In this way, four classes of ROC and AUC were obtained (Figure 9). In the ROC curve, the more convex the curve to the upper left corner, the better the model performance. The closer AUC is to 1, the better the model performance is. The AUC for individual categories in this paper reached 1, indicating that the models exhibited the desired recognition performance in the detection of these categories.

**FIGURE 10:**
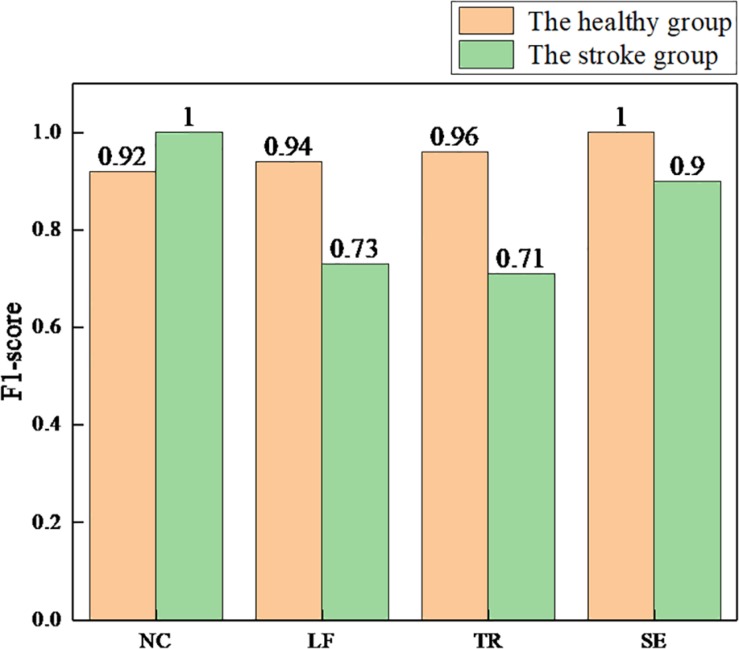
The F1-scores for each class of the health and stroke group models. NC, no compensation; LF, lean-forward; TR, trunk rotation; SE, shoulder elevation.

The F1-score is the harmonic mean of precision and recall. The closer the F1-score is to 1, the better the model performance. We used the same conversion method to calculate the F1-score per class, and the results are shown in Figure 10.

### Discussion

In this paper, the sEMG-bTCD method was proposed, and its feasibility was verified. To our knowledge, this is the first study to detect simulated trunk compensations in healthy participants and real trunk compensations in stroke participants based on sEMG signals from nine superficial trunk muscles. In addition, an active segment detection method based on the optimal SampEn threshold was proposed, and was used to detect the active segments of the sEMG signals of the healthy and stroke group, respectively. Five compound time domain features of each channel in the active segment were extracted to form the feature vector space, including RMS, VAR, MAV, WL, and 4th-ARMC. Using the SVM classifier, two four-class models for detecting three types of trunk compensations (LF, TR, and SE compensations) and NC motion were established, trained, and tested, consisting of the healthy detection model and the stroke detection model. In addition, the effect of channel number on classification was investigated, and the results showed that the best accuracy was achieved for both detection models based on nine channels. Therefore, only the experimental results of nine channels were analyzed below.

First, the healthy detection model was established based on the sEMG signals of the healthy group. The testing results showed that >90% accuracy of each class (average 95.0%) was achieved. Compared with the accuracy of 88.6 (Ranganathan et al., 2017) and 85.9% (Taati et al., 2012), higher detection accuracy was obtained by using the method proposed in this paper. We also selected the AUC and the F1-score as model evaluation parameters. The best detection performance was obtained on the SE compensation (AUC = 1.00, F1 = 1.00), followed by the TR compensation (AUC = 0.99, F1 = 0.96), and finally the LF compensation (AUC = 0.97, F1 = 0.94). When compared with the results (SE: AUC = 0.66, F1 = 0.07; TR: AUC = 0.77, F1 = 0.57; LF: AUC = 0.98, F1 = 0.82) (Zhi et al., 2017), the detection performance of SE and TR compensation in this paper was significantly improved, except for equivalent detection performance of LF compensation.

In addition, with the same process as the healthy group, the stroke detection model was established based on the sEMG signals of the healthy group. The results showed that the average accuracy of this model was 83.1%. Specifically, high accuracy (over 90%) was achieved in NC and SE compensation, followed by LF compensation (74.8%), and finally TR compensation (67.1%). Using the AUC and the F1-score to evaluate the model, the results showed that the NC detection performance was the best (AUC = 1.00, F1 = 1.00), followed by SE compensation (AUC = 0.98, F1 = 0.90), then LF compensation (AUC = 0.90, F1 = 0.73), and finally TR compensation (AUC = 0.85, F1 = 0.71). Similarly, nine stroke subjects were recruited to participate in the experiment (Zhi et al., 2017). Compared with their results (SE: AUC = 0.27, F1 = 0.07; LF: AUC = 0.77, F1 = 0.17; TR: AUC = 0.81, F1 = 0.27), it was found that the AUC and F1 values for the three types of trunk compensation are generally higher.

Overall, using the sEMG-bTCD method, we obtained better TCD performance in both the healthy and stroke groups. The results suggested the feasibility and effectiveness of this method. However, we found that TCD performance of the stroke detection model was generally lower than those from the healthy group. There may be many reasons for this result. First, actual trunk compensations in the stroke group are the joint motion of multiple muscle groups, which makes it more difficult to distinguish trunk compensations. Second, grasping the stick on the wooden flashboard for rehabilitation training, the stroke patients use the proximal (shoulder) muscles to assist because of the weakness of the distal (wrist) muscles. This action makes the DT muscle abnormally activated in various rehabilitation training tasks not just in the SE compensation. In addition, despite skin pretreatment, the collection of sEMG signals (especially from LRA, RRA, LOEA, and ROEA muscles) was affected by sensor location and soft fat tissue of human body. This observation may be one of the reasons for the low detection performance of LF and TR compensation. Moreover, stroke patients who recover better or are slightly injured have enough motion ability to produce less compensation, which is not conducive to the detection of trunk compensation. Finally, the motor strategies of the stroke patients cannot be precisely controlled may lead to simultaneous multiple trunk compensations rather than a single type of compensation simulated by the healthy group.

Future work should focus on improving the detection performance of stroke patients. Given that stroke patients may perform multiple trunk compensations at the same time, a multi-label classification model will be established, trained, and tested. In addition, a closed-loop approach, such as the use of a slider rail mechanism rather than an open-loop wooden flashboard, can reduce or even eliminate the effects of the stick on the proximal muscles of stroke patients. What’s more, multiple sensors, such as sEMG, cameras, and inertial sensors, should be fused for TCD. Finally, although lower detection accuracy was achieved with fewer channels, we will try to adopt some new methods, such as deep learning to ensure accuracy while reducing the number of muscles.

Future work should also realize the potential medical value of the proposed method and provide feedback for postural correction. Studies have shown that sEMG signals can be used for quantitative assessment of muscle spasticity (Zhang et al., 2019) and as feedback control for robot (Koh et al., 2017) or prosthesis (Zhai et al., 2017). Therefore, the future work should use sEMG signals for quantitative assessment of trunk compensations and as a feedback control for robotic rehabilitation training to correct posture.

## Conclusion

In this paper, we proposed the sEMG-bTCD method and investigated the feasibility of the method. The healthy group (five subjects) and stroke group (nine subjects) were recruited to participate in the experiment. All subjects performed three rehabilitation training tasks. The sEMG signals from nine superficial trunk muscles were collected during three rehabilitation training tasks without compensation and with three common trunk compensations. Preprocessing like filtering, active segment detection was performed and five time domain features were extracted. The four-class model obtained by using SVM classifier has excellent detection performance in healthy participants (LF: accuracy = 94.0%, AUC = 0.97, F1 = 0.94; TR: accuracy = 95.8%, AUC = 0.99, F1 = 0.96; SE: accuracy = 100.0%, AUC = 1.00, F1 = 1.00). Good detection performance was also achieved in stroke participants (LF: accuracy = 74.8%, AUC = 0.90, F1 = 0.73; TR: accuracy = 67.1%, AUC = 0.85, F1 = 0.71; SE: accuracy = 91.3%, AUC = 0.98, F1 = 0.90). The results indicate that the sEMG-bTCD method is feasible. This method helps to prompt the patient to correct the wrong posture, thereby improving the effectiveness of rehabilitation training. To enhance the detection performance in stroke patients, the compound trunk compensation should be detected instead of a single trunk compensation. Additionally, multiple sensors, such as sEMG, cameras, and inertial sensors, should be fused for TCD.

## Data Availability Statement

The raw data supporting the conclusions of this manuscript will be made available by the authors, without undue reservation, to any qualified researcher.

## Ethics Statement

Ethics approval and consent to participate (i.e., written informed consent) was obtained from all the participants to complete the protocol approved by the Guangzhou First People’s Hospital Department of Ethics Committee. All the research was performed in accordance with the Declaration of Helsinki.

## Author Contributions

KM conceived the research and participated in the entire research process, including experiments, data processing, results analysis, and manuscript drafting and revision. YC and LX conceived the research, and participated in experiments and revisions of manuscripts. XZ and HZ participated in the experiments and analysis of the results. SY and SC participated in the experiments and data processing.

## Conflict of Interest

The authors declare that the research was conducted in the absence of any commercial or financial relationships that could be construed as a potential conflict of interest.
